# Contraceptive utilization and associated factors among youths in Hossana town administrative, Hadiya zone, Southern Ethiopia

**DOI:** 10.1371/journal.pone.0275124

**Published:** 2022-11-17

**Authors:** Getachew Ossabo Babore, Asnakech Zekiwos Heliso

**Affiliations:** Department of Comprehensive nursing, School of Nursing, College of medicine and health science, Wachemo University, Hossana, Ethiopia; Flinders University, AUSTRALIA

## Abstract

**Background:**

In low-income countries out of, 60.7 million unintended pregnancies, 19% of them are subjected to abortion of which 11% of were unsafe. Surprisingly, about 2.5 million occur in women under the age of 20 years. **Aim of this study** is to measure the level of contraceptive utilization and associated factors among youths in Hosanna town administration.

**Method:**

Institutional based cross-sectional study was conducted in Hossana town administration, Hadiya zone, Southern Ethiopia. A multistage sampling procedure was employed by clustering health facilities into reproductive health clubs and health facilities to select 781 study participants. Data was collected by using structured pre-tested, self-administered questionnaires. All coded and cleaned data were entered into EPI-info version 3.5.1 and it was exported to SPSS version 16.0 for recoding and further analysis.

**Result:**

Among youths who had been sexually active within the last 12 months, 67.6% had used contraceptives prior to the survey. Multivariate analysis was found statistically significant association between contraceptive utilization and education status of mothers who attained university AOR = 4.57 [95% CI (1.29, 16.19)], utilization of sexual and reproductive health services within last 12 months AOR = 2.26 [(95% CI: 1.33, 3.86)], age initiation of first sex between 15–19 year OR = 2.63 [(95% CI 1.48,4.64)], discussion with sexual partner AOR = 1.99 [(95% CI: 1.27, 3.13)], good knowledge on contraceptive advantage AOR = [1.89 (95%CI: 1.07, 3.32)]. Whereas educational status: being secondary level decrease utilization of contraceptives by 51% AOR = 0.49 [95% CI (0.27, 0.94)].

**Conclusion & recommendation:**

The findings of our study imply that level of contraceptive utilization is higher than as compared to the previous studies. Discussion with a sexual partner as well as with a spouse, having awareness on contraceptive advantages, early age initiation of sexual intercourse, maternal educational status and getting sexual and reproductive health services recently were identified as predictors of contraceptive utilization.

## Introduction

Youths are the segment of the population where they begin actively exploring their sexuality. Also, a transition period is characterized by significant physiological, behavioral, psychological, emotional, and social changes [1, 2]. World Health Organization (WHO) defined the age groups of 10–19, 10–24 and 15–24 years as adolescents, young people and youths, respectively [3, 4]. Globally, youths constitute more than 20% of the total population. Surprisingly, 85% of them have lived in developing countries whereas, they constitute 20% and 33% out of the total population in Sub Saharan Africa (SSA), Ethiopia respectively [5–7].

In fact, a transition period is risky in addition many youths in the world often lack knowledge on: Sexual and reproductive health (SRH), the consequences of unprotected sex, and methods of contraceptives. Moreover, they have experienced less access to affordable, accessible and equitable health services. So, they are a vulnerable group of the population [8–10]. However, youths have a fundamental right to access family planning (FP) they have been facing enormous problems. For instance, unwanted pregnancies, that result in unsafe abortion and stigmatization from family [11, 12]. Girls under the age of 15 years are 5 times at high risk of dying from complications related to pregnancy or childbirth. Similarly, girls within the age range of 15–19 years as compared to 20s are 7 times higher risk of acquiring Human Immune Virus (HIV) than boys. Thus, it is important to note that early sexual debut, early marriage especially in the rural area, and very-low use of contraceptives have been associated with unwanted pregnancy [13, 14].

Globally, 2.7 million infant deaths and the loss of 60 million healthy lives were prevented by the utilization of modern contraceptives [15]. Scaling up the utilization of modern contraceptive through decreasing unmet needs is area of interest. Thus, meeting unmet needs; would avert 52 million pregnancies annually worldwide, would reduce the number of unplanned birth in developing countries by 72% as well the number of induced abortions by 64%, furthermore expected to avert 12,800 maternal & about 1.1 million child deaths [16, 17]. Therefore, investing in contraceptives delivery is beyond noticeable prevention of unplanned pregnancy and reduction of disease burden [18].

In Ethiopia, since it launched FP service was highlighted as one of the basic strategies for reducing the high risk of pregnancies, which occurs as a result of too: early, late, and frequent pregnancies. However, in Ethiopia FP service begun in 1966 yet, its coverage and utilization level by young people in the existing health care system has been seen reported as very low. [19, 20].

Globally, out of 250 million pregnancies each year one third is unintended or unwanted, of which 20% end up in induced abortion. Whereas in low- income countries from 182 million pregnancies 60.7 million is unintended, subsequently 19% are subjected to abortion out of this 11% are unsafe abortion. Surprisingly, 2.5 million occurred in women under the age of 20 years [9, 21]. Evidence has shown that out of 33% of hospital care that provided for complications occurred as a result of abortion for the woman was age bellow 20 years. Furthermore, out of total women who seek medical care, 67.2% of them treated for incomplete abortions are under the age of 24 years [22].

The consequence of unwanted pregnancy is beyond unsafe abortion as well as its complications. Moreover, accelerate rises of the fertility rate, unless substantial and multidimensional effort is undertaken, world population will grow to 9 from 7 billion whereas African population from 1.1 to 2.4 billion by 2050 [23, 24]. Besides total fertility, for many countries, adolescent fertility becomes a public concern and global agenda. Because it is associated with adverse maternal and child health outcomes includes obstructed labor, low birth weight, high infant & maternal mortality [25].

Youths’ contraceptives utilization is influenced by multiple factors; having higher education increase contraceptive utilization by 9 folds [26, 27]. Similarly, the presence of Health facilities with different set-ups, having SRHs provision facilities near to the residence, and design of service provision facilities [28, 29]. In addition, Lack of information, social stigma, judgmental attitudes by service providers, lack of confidentiality, logistics and policies had been identified as barriers to access contraceptives [30].

Despite the fact, substantial increase in contraceptive use one-third of young women age 15–24 years have an unmet need for FP and their unmeet need was twice as compared to others. In Ethiopia the contraceptive prevalence rate (CPR) among young women increased substantially from 15% - 29% in 2005 & 2011 EDHS, and the unmet need for young people is still high. It is reported as 32%, 22% among youths age 15–19 and 20–24 years respectively [31] According to the further analysis of three years EDHS 1/4^th^ of all pregnant adolescents and young women age (15–24) years feel that their pregnancies were mistimed or unwanted. Furthermore, in Ethiopia 28% of adolescents (15–19), 24% of young women (20–24) years were experienced with unintended pregnancies [31–33].

In the study area, youths account for 20% of the total population. Here are noticeable migration of young people from rural to urban for job opportunities, academic-related issues and preference of resident. But public-sector concerns and non-governmental involvement, through availing youth’s attraction centers, youth-friendly service offering facilities has been seen very few. As a result, they have been exposed to numerous SRH problems [HTA, health office report, 2016].

**Fig 1:**
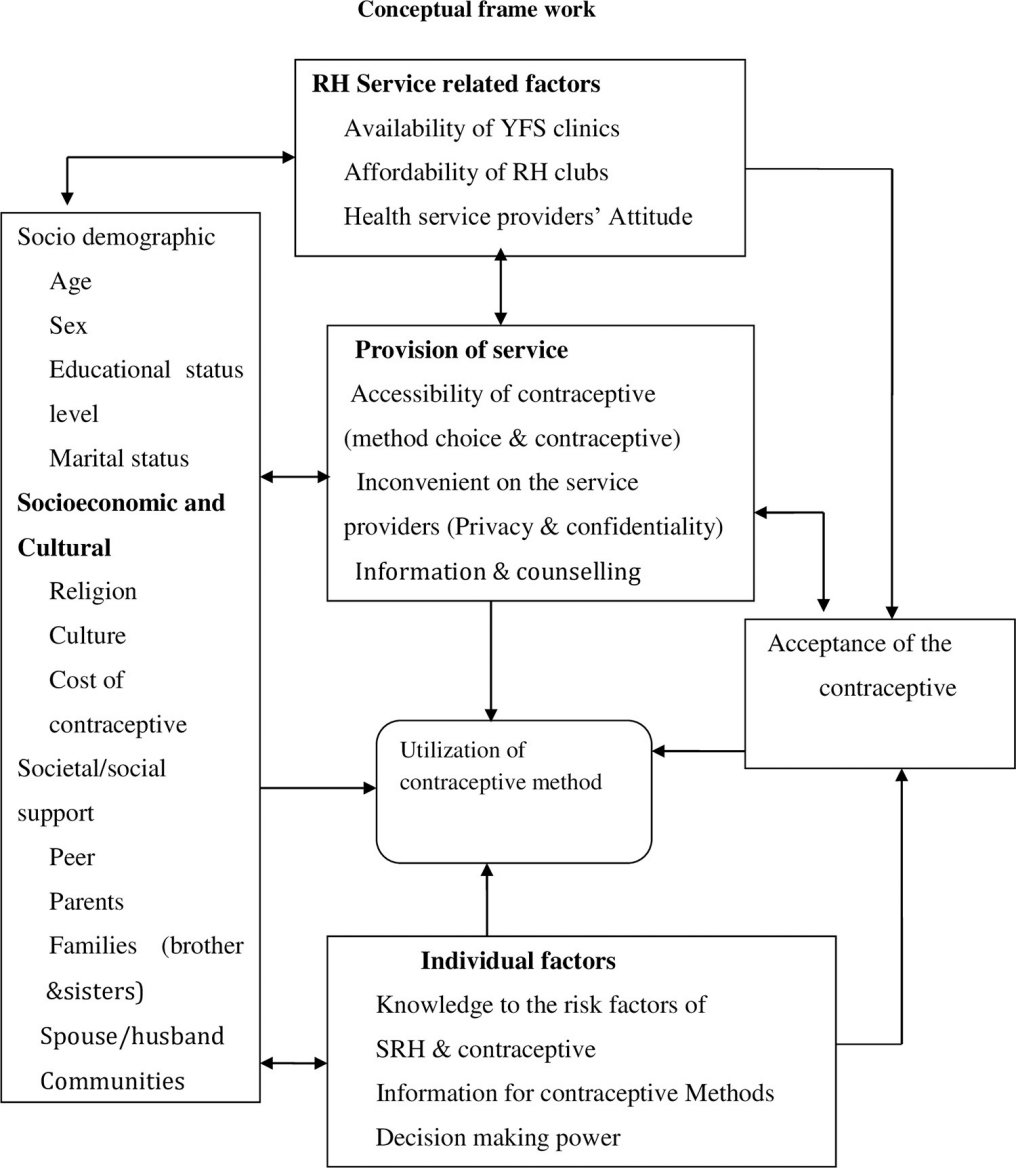
Conceptual frame work of factor affecting contraceptive utilization of youths, developed from literature review. SRH = Sexual and Reproductive Health, YFS = Youth Friendly Service, RH = Reproductive Health.

Therefore, provision of integrated SRH service under the care of healthcare professionals and investing in youths is one of the key strategies to overcome the burden on the entire population and interrupt intergeneration effect [34, 35] [Fig 1: Conceptual frame work].

## Methods and material

### Study area

The study was conducted in Hossan town administration, Hadiya Zone, SNNP regional state, Ethiopia. Hadiya zone is one of 14 Zones and four special woredas found in the SNNPR state. It has ten woredas and two towns administrative. According to the Central Statistical Agency, the total population of the zone was 1,547,848 out of these 782,128 are male whereas 765,720 of them are female [41-unpublished]. Hossana town administrative (HTA) is an urban setting, it is located in the northern part of SNNP regional state 232 KM far from the country’s capital city (Addis Ababa) and 120 KM from the regional capital town. It has 3 sub-city administrates which consists 10 kebeles (last administrative unit). According to the HTA office current budget year (2017 & 2018) projection estimation, the total population of the town administration is 100,081 out of them, youths comprise 19,286. The town administrative has one referral Hospital, three health centers (HC), one higher clinic, more than 15 Private clinics, 17 drug vendors and 5 reproductive health clubs (RHCs).

### Study design and period

Institutional based cross sectional study design, which was conducted from January 2-30/2017.

### Source and study population

The source population for this study compress all youth between the ages of 15–24 years who visit health facilities and RHCs. Whereas, Study participants consist youths’ who were visited randomly selected HC and RHCs for medical care as well as reproductive health services selected by using systematic sampling method.

### Eligibility criteria

All youths (15–24) years old visited HCs and RHCs for medical/ RH care included, but youths: who were severely ill (unable to respond as a result of a critical medical condition) as well as mentally ill, who weren’t living in HTA for the last six months, and had got training on youth friendly service/ peer education were excluded from the study.

### Sample size calculation for specific objective one

The sample size for the first specific objective was calculated by using a single population proportion formula through considering the following assumption: a confidence level of 95% with the corresponding value for the normal distribution of Zα/2 for α = 5% is 1.96, margin of error (d) = 5%, design effect (D) = 2 and considering the proportion of contraceptive utilization rate (P) among youths = 62.2% from the study [11]. Then an estimated sample size calculated by using the following statistical formula was 723.


$$n=\{\frac{{\left(Z_{\alpha /2}\right)}^{2*}P\;(1‐P)}{d^{2}}\}*D$$

After adding 10% to compensate a possible non-responses rate, the needed sample size was 795.

Also, the sample size for the first specific objective was calculated by using the statistical software Open Epi version 3.1/ Statcalc. Through considering the above assumptions, contraceptive prevalence rate 62.2% and using a total population (youths) in the study area 19,286 yield 710 samples, but after adding 10% non-response rate a sample was 781.

### Sample size calculation for second specific objective

For the second specific objective, sample size calculation was performed by using Open Epi software version 3.1. Based on the study [8] taking factors that have a significant association with contraceptive utilization are educational status, parent monitoring and living with whom parents. Then, assuming having a formal education, high parenting monitoring and living with one parent as an exposure factor with respective proportion, odds ratio and considering 95% CL, 80% power of the test (Table 1).

**Table 1:** Sample size calculation for specific objective two, factor associated with contraceptive utilization among youths.

Factors associated with contraceptive utilization	Percent of control exposed	Confidence level 1-α or CI 95%	Power or 1-β	Ratio control to case	AOR	Sample size (n)	NR-rate (10% of n)	Final sample size
Having formal educational	41.8%	95%	80%	1:3	2.4	288	29	317
High parent monitoring	12.09%	95%	80%	1:2	1.84	660	66	720
Living with parents	38.7%	95%	80%	1:5	1.6	614	61	675

AOR = Adjusted Odds ratio, CI = Confidence Interval, n = Estimated sample size, NR = None Response rate

Among the samples size calculated for factors or second objective, we had picked the largest sample size 720. Finally, by comparing sample sizes calculated for both specific objectives we had chosen the first objective sample size value, n = 795.

### Sampling procedure

Multi-stage sampling was applied to select study units by clustering facilities into two strata as RH club and HFs. Among nine health facilities, six were selected by using a simple random sampling, through applying the lottery method. These health facilities were preferred over the community because most of the time youths are around there as of having a library, youth-friendly service, a few recreation centers and high peer’s density.

**Fig 2:**
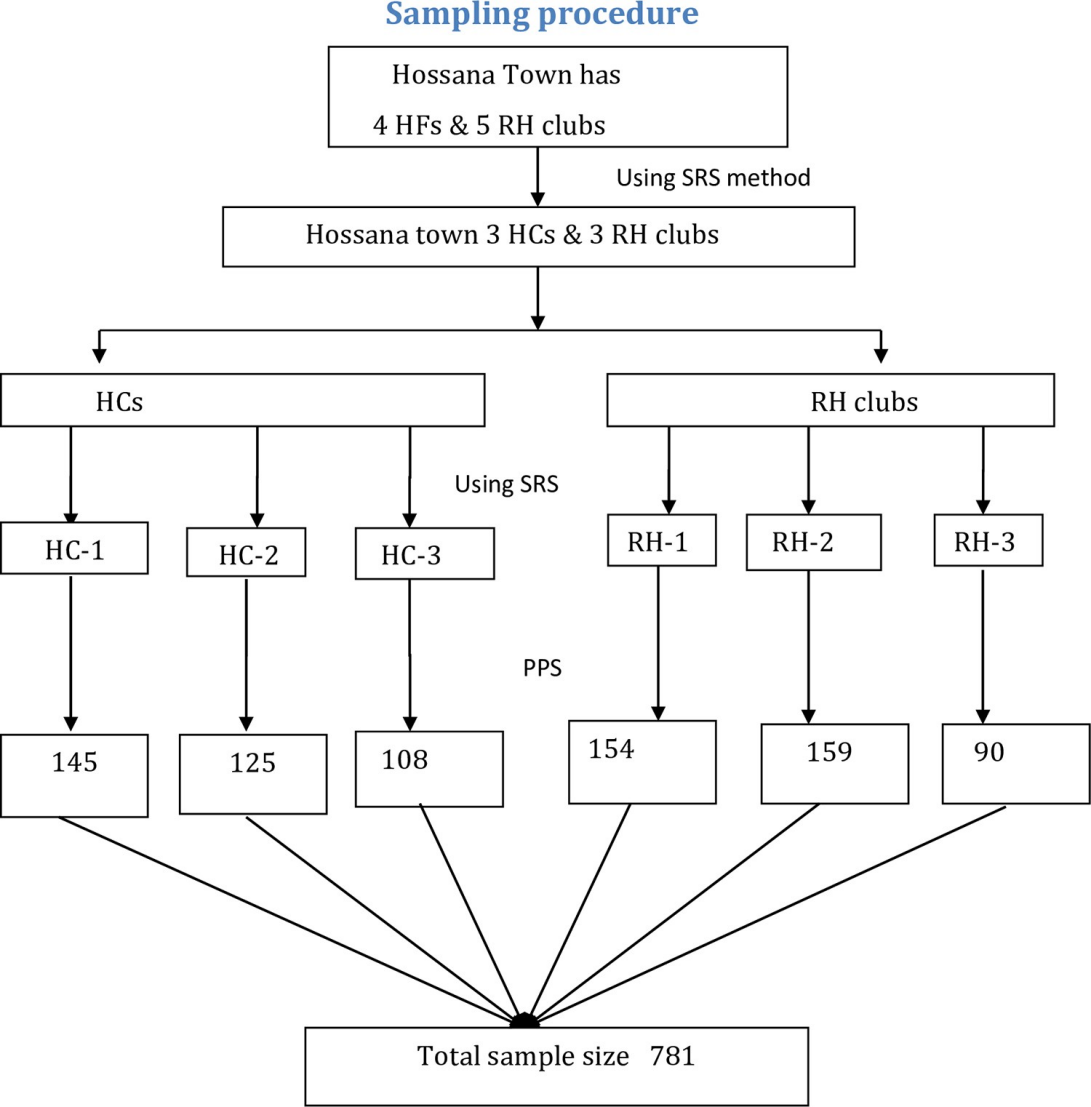
Schematic presentation of sampling procedure, contraceptive utilization among youths. RH = Reproductive Health, HFs = Health Centres, SRS = Simple Random sample, PPS = Proportionate to Population Size.

Ahead of allocating proportional size for each facility, a total number of youths, who were served in all randomly selected facilities during the last two months (N = 1807) was taken from registration books as a sampling frame. First, a total estimated sample size (n = 795) was divided by the total number of youths served during the last two months [N] in all six facilities, which gave a proportionate value (P). Secondly, proportionate value [P], multiplied by a total number of youths [Nj] those served during the last two months in each selected facility. Then, a proportionate to population size (PPS) value was provided for each facility. Finally, data were collected by using a systematic sampling method, through considering K^th^ value for each facility (**Fig 2: Sampling procedure**).

### Data collection tool, quality control and management

Data collection tool was prepared in English and translated into the Amharic language; it also back-translated to English by another translator. A tool was adapted from different kinds of literatures used in this study. Before, one-day orientation was provided for all supervisors and data collectors regarding the overall procedures of the data collection issues.

To validate the appropriateness of the tools, a pre-test questionnaire was applied among 39 youths in Gimbichu HC. After identifying difficult and unfit questions for software as well as made confusion for respondents, a necessary amendment was undertaken. Data were collected by six health professionals (clinical Nurse, Midwifery) and two supervisors under the overall coordination of the principal researcher using pre-tested structured, self-administered questionnaires. Ahead of distributing questionnaires, all ethical issues were addressed and verbal consent was taken from all voluntary youths. The research assistant and researcher had mad cross-checked for completeness, consistency & accuracy on a daily basis.

### Variables

#### Dependent variable

Contraceptive utilization.

#### Independent variables

*Socio-demographic*. Age, sex, educational status (youth), marital status, religion and educational status (parents).

*Socio-economic and cultural*. Cost to buy contraceptive, income, living arrangement, culture, sexual partners, peer (pressures), spouse.

*Health facilities related*. Distance of health facilities, availability of RH clubs, service providers’ attitude, privacy, affordability of contraceptive.

*Individual factors*. Contraceptive knowledge, practice, awareness on SRH service provision, SRH knowledge and its risk factors and decision-making power.

### Operational definition

#### Youths

Person within the age group 15–24 years.

#### Contraceptive

Any type of hormonal or non -hormonal birth control methods used by youths.

#### Contraceptive utilization

Any type of contraceptive method used at last time of sexual intercourse with in the 12 months before conducting this study.

#### Sexually active

Youths who ever had at least one penetrative sexual experience in his/her life.

#### Practice

Youths who have ever used any hormonal or non-hormonal type of contraceptive method correctly and consistently.

### Statistical analysis

All cleaned and coded data were entered into EPI-info software version 3.5.1. Then, it was exported to SPSS software Version 16.0 for further analysis and recoding. Frequency tables and cross-tabulation were used to filter errors and missing values as well as to elaborate descriptive variables.

To identify candidate variables for multivariable analysis, bivariate logistic regression was employed for each variable. Variables which have statistically significant at P value < 0.25 enter into the last model. To determine predictors of contraceptive utilization by youth’s multivariable analysis was performed. Predictor variables were identified by looking odds ratio at 95% of confidence level and p-value < 0.05.

### Ethical consideration

The study protocol was approved by the institution review board of the Woliat Sodo University/WSU whereas ethical approval and clearance letter was obtained from the institution’s review board of college of medicine and health science, WSU. Cooperation and permission letter was written for respective sectors from government offices. After all, ethical issues settled before conducting study or administering questionnaire. In addition, for those youths bellow 19 years who selected randomly parent as well as guardian permission was requested before distributing self-administered questionnaire.

All data collectors addressed and informed participants about the purpose of study and benefit. Then they assured issue of confidentiality, having full mandate to withdraw from study and refuse to participate. Finally, oral consent was taken from each participant. There is no any legal as well ethical restriction on sharing this article data. The article didn’t contain sensitive issues of human being like tissue as well as any organ. For further enquires you can communicate the institution review committee member. They witness their agreement by orally as stating I, participant bellow understands the benefit and confidentiality of this study and I agreed to participate on the study. Files documented and precede self administered questions if they agreed and otherwise we move to next participants.

## Result

### Socio demographic characteristics of the respondents

A total of 758 youths were returned self-administered questionnaires which gave a response rate of 97%. More than three fourth of participants were within the age range of 20–24 years. The majority of the respondents were Hadiya 358 (47.2%) and followed by Amhara 125 (16.5%). Nearly fifty percent of youths’ educational status was between 9^**th**^ –12^**th**^. But, only 10.9% of mothers and 26.4% of fathers reached college or TVET level. However, more than one fifth 159 (21%) of the respondents were married, 71 (9.4%) of youth living together (Table 2).

**Table 2:** A Socio demographic characteristic of youth’s at Hossana town, Southern Ethiopia, 2017.

Variable	Category	Frequency		Total	Percentage
		HC	RHC		
Age (year) (N = 758)	From15-19 years	115	66	181	23.9
	From 20–24 years	249	328	577	76.1
Sex (N = 758)	male	143	165	308	40.6
	Female	220	230	450	59.4
Religion (N = 758)	Orthodox	105	116	221	29.2
	Protestant	165	211	376	49.6
	Islam	50	34	84	11.1
	Catholic	38	29	67	8.8
	Others *	5	5	10	1.3
Ethnicity (N = 758)	Hadiya	153	205	358	47.2
	Gurage	56	38	94	12.4
	Amhara	76	49	125	16.5
	Silte	27	16	43	5.7
	Kembeta	41	54	95	12.5
	Others **	10	33	43	5.7
Educational status (respondents) (N = 758)	No formal education	4	2	6	0.8
	From 1–4 grade	9	9	18	2.4
	From 5–8 grade	62	50	112	14.8
	From 9–12 grade	166	207	373	49.2
	College and above	123	126	249	32.9
Occupation (respondent) (N = 758)	Student	198	233	431	56.9
	Employee	56	62	118	15.6
	Daily labor	18	17	35	4.6
	Merchant	58	60	119	15.7
	House wife	20	20	40	5.3
	Farmer	3	1	4	0.5
	Others ***	10	11	11	1.5
Education status (mother) (N = 758)	No formal education	102	113	215	28.4
	Primary (1-8^th^)	98	131	229	30.2
	Secondary (9-12^th^)	92	95	187	24.7
	TVET or college	52	31	83	10.9
	Higher (university)	19	24	43	5.7
Education status (Father) (N = 758)	No formal education	32	25	57	7.5
	Primary (1-8^th^)	38	66	104	13.7
	Secondary (9-12^th^)	112	132	244	32.2
	TVET or college	105	95	200	26.4
	Higher (university)	76	76	152	20.1
With whom you live (respondent) (N = 758)	Both parents	95	157	252	33.3
	One parent only	50	53	103	13.6
	Sister or brother	56	38	94	12.4
	Lonely	101	90	191	25.2
	Spouse	59	49	108	14.3
	Others*+	3	7	10	1.3

* others = seventh day, John witness and pagan ** others = Oromo, Tigre, sidama and Woliata **** = drivers *+ = with others relatives. HC = Health centre, RHC = Reproductive health club.

### Sexuality and contraceptive utilization

Among the study subject, nearly three quarters 71.6% were sexually active, out of them 316 (58.2%) were initiate sexual intercourse before celebrating 20^th^ birth date. Among sexually active youth, 385 (70.9%) of them had discussed with their sexual partner but, 5.3% of them didn’t support utilization of contraceptives. Descriptive finding also reported that thirty-four Percentage of respondent had used contraceptives while having their first sexual intercourse. Nearly three-quarters (73.8%), 32.6% of them were used condoms and ECP respectively when had first sexual contact. Youths who initiate sex below 19 years were less experienced in using contraceptives than 20–24 years. Surprisingly, only 26.4%, 36.2% of youth whose age below 19 years and 20–24 years used contraceptives respectively when had fist sex. Half of the youths who didn’t use contraceptives at first sex, reported that the main reasons were at first sex contraceptive is not necessary, had no contraceptive information at a time, and a few of them reported that it decreases sexual pleasure.

The majority, 97% of the respondents knew where they could obtain contraceptives. Almost all of the participants 743 (98.0%) reported that they had ever heard about a contraceptive. They reported as 75.6% from TV and the least 5.1% from parents by mentioning at least one contraceptive method. The most commonly reported methods were condoms 93.1%, followed by ECP 52.6% and DMPA 48.2%. However, 4.5% of respondents knew all hormonal contraceptives, but only 9.5% knew one method whereas 16.0% knew four contraceptive methods. In addition, a study assessed the benefit of contraceptives reported by youths. 56.5%, 84.6%, 50.8% of youths reported as it’s important to protect from STIs, prevent unwanted pregnancy, and prevent early pregnancy & childbirth, respectively. But the least, 2.8% of them to get money/ incentives.

### Contraceptive utilization among youths

**Fig 3:**
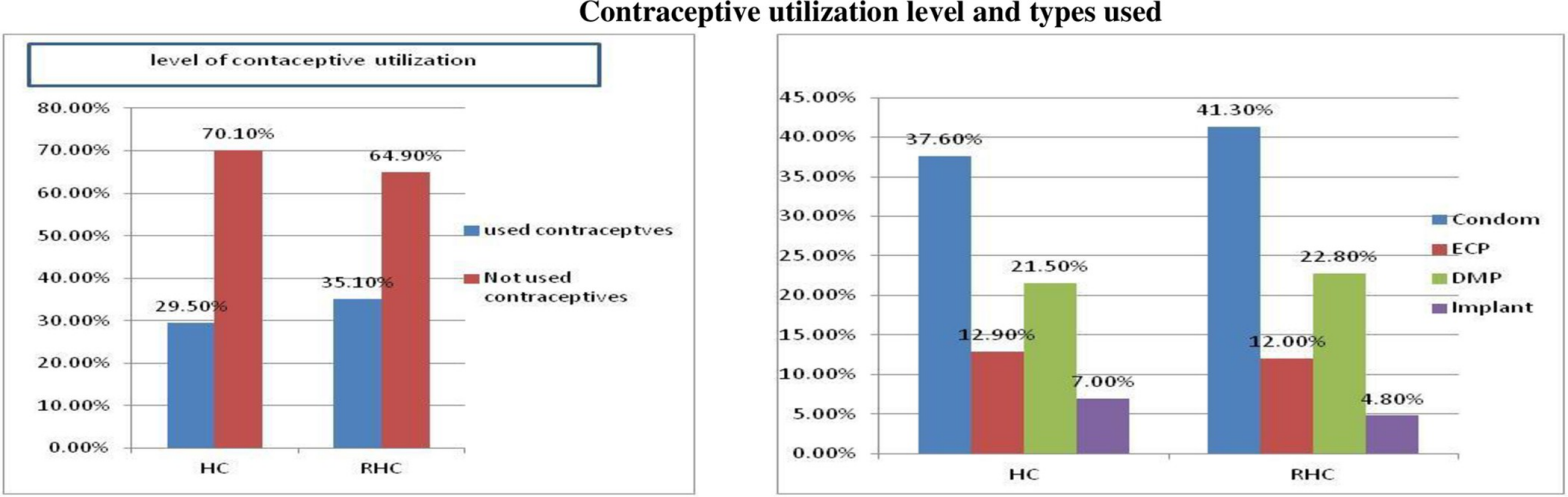
Contraceptive utilization level and types of method used, in Hossana town administration, among youths who visits health facilities, Southern Ethiopia, 2017. HC = Health centre, RHC = Reproductive health club, ECP = Emergence contraceptive pills, DMPA = Depo medroxy Progesterone Acetate.

The overwhelming majority of sexually experienced youths, 353 (67.6%) reported that had ever used the contraceptives method at the last time they had sex. The most commonly used types were condoms, 139 (39.4%), followed by ECP 73 (20.7%) and injectable 72 (20.4%) **(Fig 3: level of contraceptive utilization and types used).**

Among the respondents, more female youth 222 (68.1%) used contraceptives following the last sexual intercourse as compared with males. Similarly, those who had gotten any SRH service within 12 months before the survey used contraceptives more than their counterparties. Moreover, this study found that youths who discussed with a sexual partner or spouse 277 (74.3%) were used contraceptives more than hadn’t discussed 75 (51.7%). Participants who did not use contraceptives while having last sexual contact reported the main reasons: 57.5% lack of privacy, 54.6% fear of side effects and 28.2% refusal by a sexual partner.

### Determinants of contraceptive utilization

Bivariate logistic regression was employed to determine factors which are statistically significant with 95% of CL at P-value < 0.25. Variables: youths educational status, mother’s educational status, having a discussion with a sexual partner or spouse, youth knowing where they can collect contraceptive, contraceptive knowledge, utilization of SRH within the last twelve months, living style, age in which initiation of first sex, and youths thought that lack of money can hinder using contraceptive have been seen statistically significant.

Multivariable analysis, considering the variables which were a statistically significant in bivariate logistic regression was performed. Thus, the result of multivariable analysis had shown that mothers educational level being college and above AOR = 4.57 [95% CI: 1.29, 16.19], initiating sex with in the age of 20–24 year AOR = 3.12 [95% CI: 1.63, 5.97], having a discussion with a sexual partner or spouse AOR = 1.99 (95% CI: 1.27, .3.13), Utilization of SRH service within last the twelve months AOR = 2.26 (95% CI: 1.33, 3.86), having awareness on contraceptive advantages AOR = 1.89 (95% CI: 1.07, 3.32), youths educational level being secondary AOR = .49 [95% CI: 0.27, 0.94], and having a discussion with sexual partner AOR = 2.20 (95% CI: 1.36, 3.57) were identified as predictors of contraceptive utilization.

The multivariate model analysis shown that; mothers who attained higher level of education were 4 times more likely use contraceptive than unable to read and write, and youths who initiate sex at the age of 20–24 year, 3 times increase odds of utilization of contraceptive than their counterparties. Youths who obtained SRH service in the last twelve months 2 times more likely to use contraceptive than those hadn’t got SRH, having discussion with sexual partner or spouse regarding to contraceptive had significant association in the model, it increase nearly 2 times the contraceptive utilization level than did not discus, and knowing two and above advantages of contraceptive increase odds of utilization of contraceptive nearly by 2 folds whereas youths who attained secondary level of education 51% times less likely to use contraceptive (Table 3).

**Table 3:** Multivariate analysis of factors associated with contraceptive utilization among youths at Hossan town, in Southern Ethiopia, 2017.

Variable	Category	Contraceptive utilization		Crud odd ratio (COR)	Adjusted odd ratio (AOR)	P-value
		Yes	No			
Education status of respondent	Primary & below +	66 (75.8)	21 (24.1)	1	1	
	Secondary	156 (61.7)	97 (38.3)	.512 [.29, 89]*	.49 [.27, .94]**	0.01
	college/TVET to higher	131 (72.0)	51 (28.0)	.817 [.45, 1.47]	.682 [.35, 1.34]	
Educational. status of mothers	Unable to read & read+	25 (55.6)	20 (44.4)	1	1	
	Able to	69 (74.2)	24 (25.8)	2.30 [1.09, 4.87]*	3.20 [1.37, 7.47]**	0.015
	read & write	109 (69.0)	49 (31.1)	1.78 [.90, 3.51]	2.36 [1.09, 5.11]**	0.045
	Primary	84 (62.7)	50 (37.3)	1.34 [.68, 2.66]	1.58 [.73, 3.41]	0.029
	Secondary	44 (67.7)	21 (32.3)	1.68 [.77, 3.67]	1.78 [.730, 4.32]	
	Collage & TVET University	22 (81.5)	5 (18.5)	3.52 [1.13, 10.95]*	4.57 [1.29, 16.19]**	
Obtaining SRH	Yes	40 (47.1)	45 (52.9)	2.84 [1.77, 4.56]**	2.26 [1.33, 3.86]**	0.02
Service in 12 months	No	313 (71.6)	124 (28.4)		1.00	
Age of sex initiation	Less than 15 year	37 (49.3)	28 (50.7)		1.00	
	15 to 19 years	231 (70.5)	89 (29.5)	2.46 [1.47, 4.12]*	2.63 [1. 49, 4.64]**	0.01
	20–24 years	102 (72.3)	39 (27.7)	2.69 [1.50, 4.82]*	3.12 [1.63, 5.97]**	
Discussion with sexual partner or spouses	Yes	277 (74.3)	96 (25.7)	2.69 [1.09, 4.02]*	1.99 [1.27, 3.13]**	0.01
	No	75 (51.70	70 (48.3)		1.00	
Contraceptive advantages knowledge	One advantage +	29 (64.4)	16 (35.6)	1	1.00	
	Two advantages	91 (70.5)	38 (29.5)	2.25 [1.37, 3.69]*	1.89 [1.07, 3.32]**	0.014
	Three and above	229 (67.6)	110 (32.4)	1.90 [1.14, 3.18]*	1.736 [.962, 3.13]	

## Discussion

This study found, 67.6% of youths had ever used contraceptives when they had last sexual intercourse. The finding is less than studies conducted in Gondor 79.2% [28] and South Africa 89.1% [36]. But it is higher than studies done in Addis Ababa 61.8% [37], Uganda 62.2% [38] and three Bolivian cities [29]. The possible reason for low utilization could be study area setting, youths’ age difference and sexuality status. A Study conducted in Gondor, consists of youth’s age 15–19 years only and the town is more advanced than our study area, whereas South Africa study considered among sexually active youths only. Those may bring different opportunities for better awareness on contraceptives following sexual intercourse.

A substantial number of studies identifies that youths with primary and above education were more likely to utilize contraceptives. In this study 61.7% of youths whose educational status is secondary used contraceptives. This finding is less than a study done in Gondor 86%, [28] Nambia 77% [39] whereas higher than from studies conducted in Uganda 35% [38], Kenya 19.7% [40] and South Africa 59.1% [18]. The difference might be related to the educational compositions of youths’, because educated youths may have good knowledge as well as better awareness of contraceptives in turn may increase utilization of contraceptives.

Primary and above education status of mothers were increase contraceptive utilization of youths by 2 folds as compared to their counterparties. Our finding is in agreement with a study done in Gondor [28]. The fact that educated mothers more open and willing to discuss with their youths on SRH maters. In this study utilization of any SRH services recently were more likely to utilized contraceptive as compared to their counterparties. This proportion was lower than study done in Uganda 75.9% [41]. On another hand it is higher than studies done in Bahir Dar 32.2% [5] and Kenya 29.5% [40], the possible reason for the higher proportion could be our study included youths who visited RH clubs in addition to health facilities, so youths here might get more contraceptive information and counseling services from health workers, which in-turn increase better awareness on contraceptive utilization.

This study found that there is significant association between age of sexual initiation and contraceptive utilization. Out of sexual active youths’ 14.5%, they reported having had sexual intercourse before they turned 15 years of age. The finding is higher than study conducted in Dasie town, 8.2% [42], in Bishofitu, Ethiopia 12.7% [37], 6.9% in South Africa [36] and Uganda 11% [38].The possible reason for the deference could be the socio cultural prohibition of youth to report premarital sex, and youths who exposed to the different pornographic film impose them to early initiation of the sex.

Among 70.9% of youths had discussed with their spouse or sexual partner, 74% of were used contraceptive when had last sexual intercourse. Which increase odds of contraceptive utilization by 2 folds. This finding was in-line with study conducted in Nambia 62% [39], South Africa 60.7% [18]. The proportion is higher than Namibia & South Africa. The possible reason might be the analysis of Namibia was from Namibian DHS, it consists both rural and urban youths’. Rural youth due to less access to SRH service, cultural inhibition and information may have poor communication with partners.

## Conclusion

This study demonstrated that contraceptive utilization rate among youths was higher than as compared to previous studies. The finding revealed that having discussion with sexual partner/spouse, obtaining any SRH service recently, had awareness on advantage of using contraceptives and age of initiation of sexual intercourse were predictors of contraceptive utilization whereas educational level of respondents decrease odds of contraceptive utilization.

This study found that sexually active youth who had sexual exposure recently did not used contraceptive. They reported the main reasons due to fear of privacy, lack of spousal support, fear of health professionals’ attitude and fear to be seen by somebody knows.

### Recommendation

Active and early sensitization of youth in school, reproductive clubs, youth centers and youth friendly clinics through seminaries, review meeting, coffee ceremonies, peer to peer in-door and out-door play and other gathering that create an opportunities where youths share when and how the use contraceptive at any an occasional and unplanned sexual exposures. Skill development training and sensitization should be undertaken among youths to improve negotiation between sexual partners as well as spouses.

Governments, implementing partners, and other concern bodies should pay attention towards increasing the service offering facilities like, Youth friendly clinic integrating with health centers, youth centre linking with town municipal and others RH clubs. This will give comfortable zones for youths because it guarantees their confidentiality which in-turn increase access to get services and create opportunity to discuss freely with sexual partners.

The governing body (FMOH), implementing agencies should have developed strategy and guide line for better delivery of the service especially for the youths who initiate sex at late age.

## Supporting information

S1 File (DOCX)

S2 File (DOCX)
