# Supplementary material for: Contraceptive utilization and associated factors among youths in Hossana town administrative, Hadiya zone, Southern Ethiopia
Source: PLoS One. 2022 Nov 17;17(11):e0275124. doi: 10.1371/journal.pone.0275124 (PMC9671311; doi:10.1371/journal.pone.0275124)
Supplement: S2 File — (DOCX) [file pone.0275124.s002.docx]

### Annex II: Questionnaires Wolaita Sodo University School of Public Health

The following questionnaires are prepared to measure, prevalence of contraceptive utilization & associated factors affecting service utilization among youths at Hossana, in Haddiya zone, Southern Ethiopia.

on this questionnaries no need to write the name or address of the respondents. We kindly request to you the tru and right ansewr.

001 Questionnaires code

002 Study area: zone -------------------------------- Woreda --------------

Data collector name ------------------------------------------------------

Supervisor name ------------------------------------------------------

Data collected Day -----------month---------------- year---------

**Please circle your answer**

| Q S/N | Questions | | | Response for questions & coding category | Skip |
| --- | --- | --- | --- | --- | --- |
|  | **Part I Questions on socio demographic** | | | |  |
| 101 | How old are you (in year)? | | | ----- |  |
| 102 | Sex of the respondent | | | 1,.…….Male  2,..........Female |  |
| 103 | What is your religion? | | | 1,……..Orthodox  2,……..Protestant  3,………Muslim  4,………catholic  5,…….others specify |  |
| 104 | What is your ethnicity? | | | 1,………Hadiya  2,………Gurage  3,………Amhara  4,………Silte  5,……….Kembeta |  |
| 105 | What is highest grade that you have attended | | | 1,……..Grade 1- 4^th^  2………Grade 5- 8^th^  3,……..Grade 9-12^th^  4……..(collage & TVET)  5,…….University |  |
| 106 | What is your occupation?  ( current) | | | 1,…….Student  2,……Employee (gov’t or Non gov’t)  3,……Merchant  4,……House wife  5,……Farmer |  |
| 107 | What is your Mothers educational status | | | 1,……. No formal education  2,……...read and write  3,……..primary (Grade 1-8^th^ )  3,……..secondary Grade (9-12^th^ )  4,……..Technical/vocational/college and above |  |
| 108 | Father educational status | | | 1,……. unable to write & read  2,……...read and write  3,……..primary (Grade 1-8^th^ )  3,……..secondary Grade (9-12^th^ )  4,……..Technical/vocational/college  5………Higher ( University ) |  |
| 109 | With whom do you (usually ) live | | | 1,…….with my both parents  2,…….with one parents  3,…….with my sister/ brother  4,……. Alone |  |
|  | **Part II: questions on Sexual reproductive health** | | | |  |
| 201 | Do you know from where you can get sexual and reproductive health service | | 1,………Yes  2,………No | | Skip to Q 203 |
| 202 | If you say yes for **Q 201** From where you can get sexual and reproductive health service | | 1,……..Health center  2,……..Reproductive health clinic  3,……..private clinic  4,……..Hospital  5,……..Anti HIV/AIDs  6,……..school club  8,……..health post  7,……others specify --------- | |  |
| 203 | Have you got any sexual and reproductive health service during the last 12 months? | | 0,……..yes  1,……...No | | Skip to Q 301 |
| 204 | If you **yes for Q 203** Where did get you the service? | | 1,…….Health center  2,…….Reproductive health clubs  3,…….Hospital  4,…….Anti HIV/AIDs clubs  5,…….schools clubs  6,…….private clinic  7,……youth centre | |  |
| 205 | Which service you have got (recently)? | | 1,…….Voluntary & counseling test  2,…….Harm full traditional practice   counseling/education  3,…….Sexual transmitted infection   Diagnosis and treatment  4,…….contraceptive service  5,…….counseling services  6,…….health education  7,……pregnancy test | |  |
|  | **Part III: questions on sexuality and contraceptive usage** | | | |  |
| 301 | Marital status | | 1,………….married  2,………….cohabitant/living together  3………….Never married/single  4 ------------- others | | Skip to  Q 304 |
| 302 | Have you ever had sexual intercourse? | | 1,………Yes  0,………No | | Skip to Q 311 |
| 303 | How many sexual partners do you have? | | 1,………one  2,……….two  3,……….More than two | |  |
| 304 | What was your age when you had started first sexual intercourse | | 1,……….Less than 15 years  2,……….15-19 years  3,……….20-24 years | |  |
| 305 | Have you ever discussed with your spouse or sexual partner about contraceptive use | | 1,……..Yes  0,……..No | | Skip to Q 308 |
| 306 | If you say Yes for **Q 305** did your husband/ sexual partner support using contraceptive method | | 1,…………Yes  0,…………No | | Skip to Q 308 |
| 307 | If your answer is **No for Q 306** why he/she didn’t support using contraceptive? | | ----------------------------- | |  |
| 308 | Did you use any type of contraceptive method when you had sex for the first time? | | 1,…………….yes  2,…………….No | | Skip to Q 310 |
| 309 | Which method of contraceptive did you use? | | 1,……………Condom  2,…………… pills (combined oral contraceptive   pill or progesterone only pill)  3,……………EOC (emergency oral contracptive)  4,……………Depo provera (injectable) | |  |
| 310 | Why did not use contraceptive when you had first sex? | | ------- | |  |
| 311 | Do you know about any type of modern contraceptive method? | | 1,…………Yes  0,…………No | |  |
| 312 | If you say yes for Q 311 which method do you know  (you can circle more than one answer ) | | 1,……….Condom  2,……….pills (combined oral  Contraceptive/ progesterone only pill)  3,………EOC  4,………Depo provera (injectable)  5,………Implant (inplanon or Jaddel)  6,………IUCD (intra uterine device )  7,………permanent | |  |
| 313 | From whom/where have you heard about contraceptive method | | 1,……….TV  2,……….Radio  3,……….school teachers  4,……….Parents  5,……….Peers  6,……….Sisters or brothers  7,……….Health professionals  8,……..others | |  |
| 314 | Do you know where you can get contraceptive service? | | 1,……….Yes  0,………No | |  |
| 315 | What is advantage of contraceptive? | | 1,………To prevent STI infection   2,………To prevent unwanted   pregnancies  3,………To get sexual satisfaction  4,………To get money  5,………To avoid early pregnancy  And child birth  6,……….To be successful in | |  |
| 316 | Have you ever discussed with your family about contraceptive? | | 1,…………Yes  0,…………No | |  |
| 317 | If you yes for the **Q 316** with whom you have discussed? | | 1,………...Parents  2,…………Brother/ sisters  3,…………spouse (wife or husband)  4,…………Other relative | |  |
| 318 | With whom other than family members you have discussed about contraceptives | | 1,………….friends  2,………….peers group  3,………….sexual partner  3,………….health professionals  4,………….teachers  5,………..others | |  |
| 319 | Have you ever had sexual intercourse with in the last 12 months? | | 1,…………..Yes  0,…………..No | | Skip to Q 402 |
| 320 | Did you use any type of contraceptive when you had last sexual intercourse | | 1,………..Yes  2,………..No | | Skip To Q 401 |
| 321 | If you say **yes for Q 320** Which method you used when you had last time sexual intercourse or currently using? | | 1,………..Condom (male or Female)  2,………..Pill (combined oral contraceptive or   progestron only pill)  3,……….EOC (Emergency oral contraceptive)  4,……….Depo provera (injectable)  5,………..Implant (Jaddele, Implanin  6,………...IUCD (inter uterine contraceptive) | |  |
| 322 | Where did you get the contraceptives you used for the last sexual intercourse time? | | 1,………..Government health  center/Health post/ Hospital  2,………..RH clubs/clinic  3,………..private clinic  4,………..drug vender/shop | |  |
| 323 | What is your most important reason to choice health center/health post or hospital/Reproductive health clinic to collect contraceptive? | | 1,……..friendly staff  2,………No registration  3,………available in short distance  4,……….free service  5,……….presence of all choice  6,……….provision of full information  about use  7,………have special place for  youths | |  |
| 324 | Did the service providers re-assure you that any information concerning your personal situation and the service you received will remain confidential | | 1,……..yes  0,……..No | |  |
| 325 | Do you think that, the facility is appropriate to obtain contraceptive service? | | 1,……….yes  0,……….No | |  |
| 326 | Why you choice drug venders or shops | | 1,……….No registration  2,……….Doesn’t need prescription  3,……….short waiting time  4,……….affordable service  5,………..free service  6,………..fear of bad health  Workers attitude  7,……….no health facilities near to  The residence  8,……….confidentiality respect  9,……….Easy to collect contraceptive  10,………others | |  |
| 327 | Have you encounter problems while using contraceptive? | | 1,………..Yes  0,………..No | | Skip to Q 402 |
| 328 | If you say for **Q327** What problem you have faced while using contraceptive method? | | 1,……….lack of knowledge  How to use Properly  2,……….shortage of money  3,……….forgotiviness  4,………rupture of condom  5,……….long waiting  time at the health facilities  6,………disapproval  From sexual partner  7,………disapproval from spouse  8,………lack of information  Where I can get it  9,………bad health workers   attitude  10,………fear to buy | |  |
|  | **Part IV questions on factor affects contraceptive use** | | | |  |
| 401 | Do you think why you dislike using contraceptive methods following last sex? | 1,………Fear of side effect  2,……….cultural opposition 3,………religion opposition  4,……….afraid of being  Seen by parents  5,………lack of approval from  sexual partner or spouse  6,……… approval lack from spouse  7,……….I have no Information  8,……….don’t know where  I can get contraceptive  9,………fear of bad health workers  attitude  10,………..I can’t afford to buy  11,………..fear of being seen by  some-one who know me  12,…….lack of privacy  13,……….preferred source offering  14,………others | | |  |
| 402 | Do you think lack money ever hindered you from the use of contraceptive serves? | 1,………….yes  0,………….No | | |  |

**Thank you!!**
